# Modeling of inflicted head injury by shaking trauma in children: what can we learn? Part I: A systematic review of animal models

**DOI:** 10.1007/s12024-019-0082-3

**Published:** 2019-03-04

**Authors:** Marloes E. M. Vester, Rob A. C. Bilo, Arjo J. Loeve, Rick R. van Rijn, Jan Peter van Zandwijk

**Affiliations:** 1 Academic Medical Center Amsterdam, Department of Radiology and Nuclear Medicine, Amsterdam UMC, University of Amsterdam, Room G1-231, Meibergdreef 9, 1105AZ Amsterdam, The Netherlands; 2 Specialist Services and Expertise Division, Netherlands Forensic Institute, Laan van Ypenburg 6, 2497 GB The Hague, The Netherlands; 3 Department of BioMechanical Engineering, Delft University of Technology, Mekelweg 2, 2628 CD Delft, The Netherlands; 4 Division of Digital and Biometric Traces, Netherlands Forensic Institute, Laan van Ypenburg 6, 2497 GB The Hague, The Netherlands

**Keywords:** Closed head injuries, Child abuse, Forensic pathology, Animal models

## Abstract

Inflicted blunt force trauma and/or repetitive acceleration-deceleration trauma in infants can cause brain injury. Yet, the exact pathophysiologic mechanism with its associated thresholds remains unclear. In this systematic review an overview of animal models for shaking trauma and their findings on tissue damage will be provided. A systematic review was performed in MEDLINE and Scopus for articles on the simulation of inflicted head injury in animals. After collection, the studies were independently screened by two researchers for title, abstract, and finally full text and on methodological quality. A total of 12 articles were included after full-text screening. Three articles were based on a single study population of 13 lambs, by one research group. The other 9 articles were separate studies in piglets, all by a single second research group. The lamb articles give some information on tissue damage after inflicted head injury. The piglet studies only provide information on consequences of a single plane rotational movement. Generally, with increasing age and weight, there was a decrease of axonal injury and death. Future studies should focus on every single step in the process of a free movement in all directions, resembling human infant shaking. In part II of this systematic review biomechanical models will be evaluated.

## Introduction

Head injury in young children (under the age of 5 years) can be caused by several mechanisms, such as compression/crushing, blunt force, repetitive acceleration-deceleration, and penetration. These mechanisms may lead to injuries to the skin and/or skull and/or intracranial contents. The circumstances under which these mechanisms arise can be accidental (e.g. traffic accidents or falls from height) or inflicted (e.g. child abuse). The most prevalent causes of inflicted head injury in children are blunt force (IHI-BFT: inflicted head injury by blunt force trauma) and repetitive acceleration-deceleration/shaking (IHI-ST: inflicted head injury by shaking trauma) [1]. In the Western world the incidence of inflicted head injury is estimated to be 20–40 per 100.000 children under the age of 1 year and decreases with increasing age [2–8]. In literature, inflicted head injury in children has been referred to by many different terms (Table 1). Most of these terms are suggestive and imply a trauma mechanism or a certain intention. Therefore these terms should be avoided in a forensic context.

**Table 1:** Inflicted head injury by shaking trauma in children: synonyms in the medical literature

Synonym	Interpretation / perception
Shaken baby syndrome	Trauma mechanism: shaking
Shaken impact syndrome	Trauma mechanism: shaking and impact
Whiplash shaken infant syndrome	Trauma mechanism: shaking
Schűtteltrauma	Trauma mechanism: shaking
Syndrome du bébé secoué	Trauma mechanism: shaking
Skakvald	Trauma mechanism: shaking
Abusive head trauma	Intention: abusive
Non-accidental head injury	More or less neutral
Inflicted traumatic brain injury	More or less neutral
Inflicted head injury	More or less neutral

Blunt force has never been the subject of much debate as a causative mechanism in inflicted head injury. Shaking still seems to be the subject of an ongoing debate, especially in courts, despite reliable medical and biomechanical scientific evidence that violent shaking can cause severe head injuries. This ongoing debate is caused by the fact that ‘shaking’ as cause of inflicted head injury (IHI) is a conclusion that is mainly based on exclusion of other causes (medical conditions, birth trauma, and accidental trauma after birth), combined with the absence of findings consistent with blunt force trauma (e.g. bruising of the scalp or a skull fracture) and confessions of perpetrators. Because the use of human infants as experimental research population is impossible due to ethical standards, research is restricted to juvenile animals, mechanical surrogates, and mathematical models.

The aim of part I of this systematic review is to discuss juvenile animal studies used to cause intracranial and retinal injuries after repetitively induced head motions without a direct impact mechanism. Part II will elaborate on the physical and mathematical models concerning shaking in young children.

**Fig. 1:**
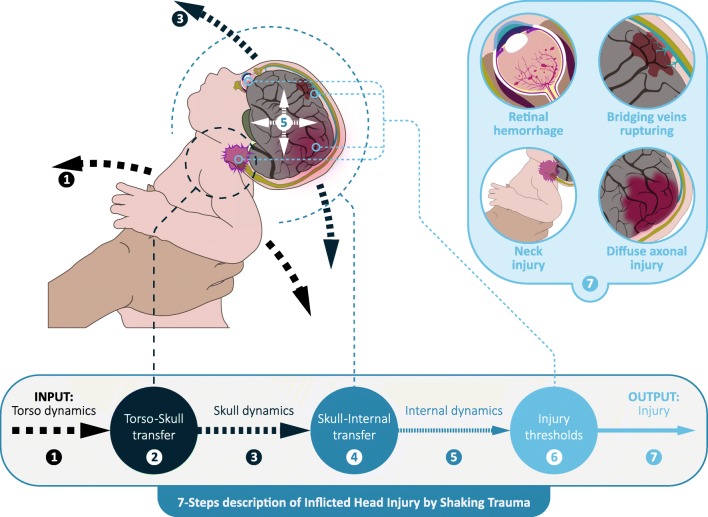
The 7-step description of inflicted head injury by shaking in children

In shaking the acceleration-deceleration forces are mostly oriented within the sagittal plane (forward-backward), but movements in the transverse (‘no’- shaking of one’s head; around the body axis, also commonly referred to as axial or horizontal) and coronal (sideways; ear to shoulder) plane will also occur. Since sagittal movements are considered to be the main component in shaking, most of the injury is also believed to be the result of this motion [9]. In shaking, forces applied onto the torso (grasping and shaking) are transferred by the neck to the skull, followed by stresses and strains on the soft tissues in the skull (Fig. 1). When stress and strain exceed certain thresholds, material failure of the tissue (injury) will occur, such as retinal hemorrhages, rupture of bridging veins leading to subdural hemorrhages (SDH), and post-traumatic encephalopathy (i.e. diffuse axonal injury; DAI). These physiological transference of forces will be discussed in more detail in Part II of this systematic review. Conclusive data on the exact pathophysiology and threshold values needed to cause these injuries in cases where IHI-ST is suspected has not been reported in the literature. The purpose of this review is to identify animal models specific for shaking trauma and their findings concerning tissue damage.

## Methods

### Database search

MEDLINE (Pubmed) and Scopus® were systematically searched up to January 1st, 2017. Five search queries were built, using both free terms and indexed terms for mechanical models, mathematical models, and animal models that mimic IHI-ST (Appendix). Articles in Dutch, English, French, and German were included.

### Article selection

Identified articles were de-duplicated in Endnote and subsequently divided into the three (physical, mathematical, and animal) study models. Two researchers (RR and MV) each assessed all articles in the animal subgroup on title, abstract, and lastly on full text, based on relevance for the understanding or explanation of (aspects of) IHI-ST pathophysiology. In case of disagreement, a third researcher (RB) was consulted. Manual reference snowballing of the included articles was performed by RR and MV. The main authors of the included articles were contacted for additional, possibly unpublished studies and information.

All prospective animal studies on the biomechanics of IHI-ST were included. Exclusion criteria were direct (blunt force) trauma to the head, non-objective studies (observational studies of animal behavior), or adult animals (because of incomparable development of the nervous system, matured muscle strength and weight). Full-texts were assessed on the methodological quality using a standardized form adapted from the Critical Appraisal Skills Program (CASP) (available upon request) [10]. In case of doubt on the methodological quality or study design, the main authors were contacted for additional information.

### Data extraction

Data extraction was performed by authors MV and RB using a predefined data-extraction form (available upon request). Baseline features such as study design and animal characteristics (e.g. age, gender) were recorded. Furthermore, trauma mechanism, measurement of inflicted forces and accelerations, cerebral macroscopy and microscopy, ophthalmological results, and the main interpretations and conclusions were extracted.

## Results

### Search results & quality assessment

**Fig. 2:**
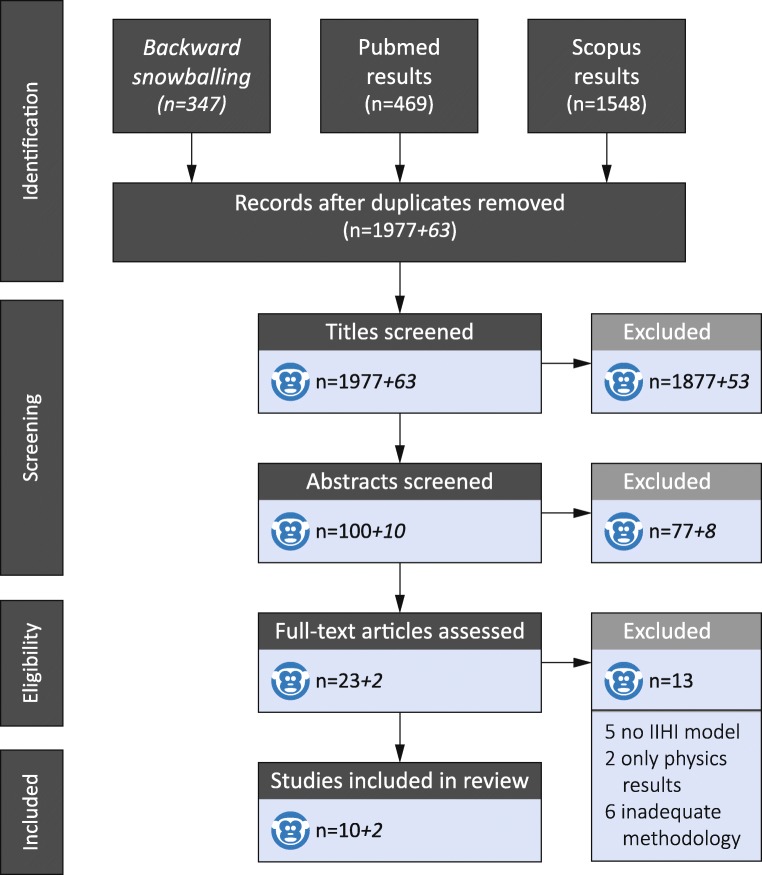
Prisma flowchart for the conducted literature search and article selection process of animal inflicted head injury by shaking. Numbers in the flowchart following the ‘+‘sign are articles identified through snowballing

The initial search resulted in a total of 4675 articles, of which 1977 eligible articles remained after deduplication (Fig. 2). 1954 articles were excluded based on title or abstract, leaving 23 articles for full-text assessment. Thirteen articles about IHI-ST were excluded based on no IHI-ST, absence of neuropathophysiological results, or inadequate methodological reporting [11–18]. Therefore, ten articles remained after full-text assessment, and two additional articles were identified by snowballing. The remaining 12 articles, three in lambs and nine in piglets, were published by two research groups. The two main researchers of these groups (JW Finnie and S Margulies) were contacted for additional information. The three articles on lambs originated from a single study, whereas the nine piglet studies were all individual studies. All studies were small, prospective studies of low quality as assessed by CASP.

### Studies in lambs

The Finnie-group published three articles on a single study design [9, 19, 20]. Table 2 provides extensive study details. The overall design included nine ‘injured’ and four control lambs, 5-to-10-days-old. The ‘free shaking’ mechanism applied by humans on the lambs in this study design, closely resembled shaking in human babies, according to the authors. Manual shaking of lambs caused extra-axial hemorrhages in both ‘younger lambs’ (5–6 kg) and ‘older lambs’ (8.5–12 kg) (Table 2). ‘Injured’ animals had significantly more β-APP positive neuronal perikaryons, equal in both age groups. Nevertheless, total injury scores and cranio-cervical junction related injury (region of maximal impact loading), hypoxic edema without ischemia, and C-fos immunoreactivity were higher in the ‘younger lambs’ compared to the ‘older lambs’ in the publications of 2012 and 2013 (Table 2) [19, 20]. None of the spinal cords showed parenchymal hemorrhages or hypoxic-ischemic damage. The first published article in 2010 did not report on the three ‘younger lambs’, which all died before the designated survival time of 6 h post-injury, with signs of Axonal injury (AI), neuronal reaction, and albumin extravasation [20].

**Table 2:** The single study design used for the three articles by Finnie et al. 2010, 2012, and 2013 [1–3]

Journal	Journal of Clinical Neuroscience
Objective	To develop a satisfactory biomechanical model for the pathogenesis of non-accidental head injury
Animals	7 lambs + 3 controls (2010 article) [1] - Age matched 7- to-10-day-old, 8.7 kg (5–12) 9 lambs + 4 controls (2012 and 2013 articles) [2, 3] - Age matched 7- to-10-day-old, 8.7 kg (5–12)group 1 (*n* = 6): ‘older’, 10.3 kg [8.5–12 kg] - Age matched 7- to-10-day-old, 8.7 kg (5–12)group 2 (*n* = 3): ‘younger’, 5.5 kg [5–6 kg] - Age matched 7- to-10-day-old, 8.7 kg (5–12)controls (*n* = 4): 7- to-10-day-old, [5–10.5 kg]
Trauma mechanism	Anesthesia and ventilation. Manually grasped under axilla, vigorously shaken, head back and forth with considerable lateral/rotational movement for 10 × 30 s in 30 min. No head impact.
Histopathology fixation	6 h full anesthesia before death by formalin perfusion fixation. Brains remained 2 h (‘overnight’ in 2012 and 2013 articles) in situ and 7 days ex-situ immersed in formalin.
Outcome measures	Macroscopy and microscopy of brains and rostral cervical spinal cord; 5 mm slices of brain and spinal cord: - β-APP immunohistochemistry and HE-staining (2010 and 2012 articles) - HE-staining, c-fos-staining, and EMA staining. (2013 article) Ocular examinations (2010 and 2012 articles)
Head and shaking kinematics	Published by Sandoz et al. 2012 and Anderson et al. 2014 [4, 5]: shaking by human subjects was applied with a frequency of about 2 Hz, thus ±40 cycles/episode. Resulting accelerations were between 40 and 80 g, with an average peak acceleration of 62 g.

β-APP immunohistochemistry: (upregulation indicates differentiation of neurons after injury), C-fos and EMA (epithelial membrane antigen): indication of neuronal activity. *HE-stain* hematoxylin and eosin stain

‘Injured’ lambs, more commonly the younger ones, showed damage of the retina with increased GFAP, multifocal damage of the inner nuclear layer neurons, mild segmental splitting, and increased β-APP expression [19, 20]. Additionally, there was albumin extravasation in the uvea. Minor retinal hemorrhages were, amongst others, seen in both of the ‘older lambs’ with a SDH [9].

Overall, the ‘injured’ lambs showed injuries of the brain, spinal cord, and eyes, while the control animals did not show any relevant abnormalities. Injury was more common and more extensive in the lower weight, younger lambs, which all died prematurely.

### Studies in piglets

**Fig. 3:**
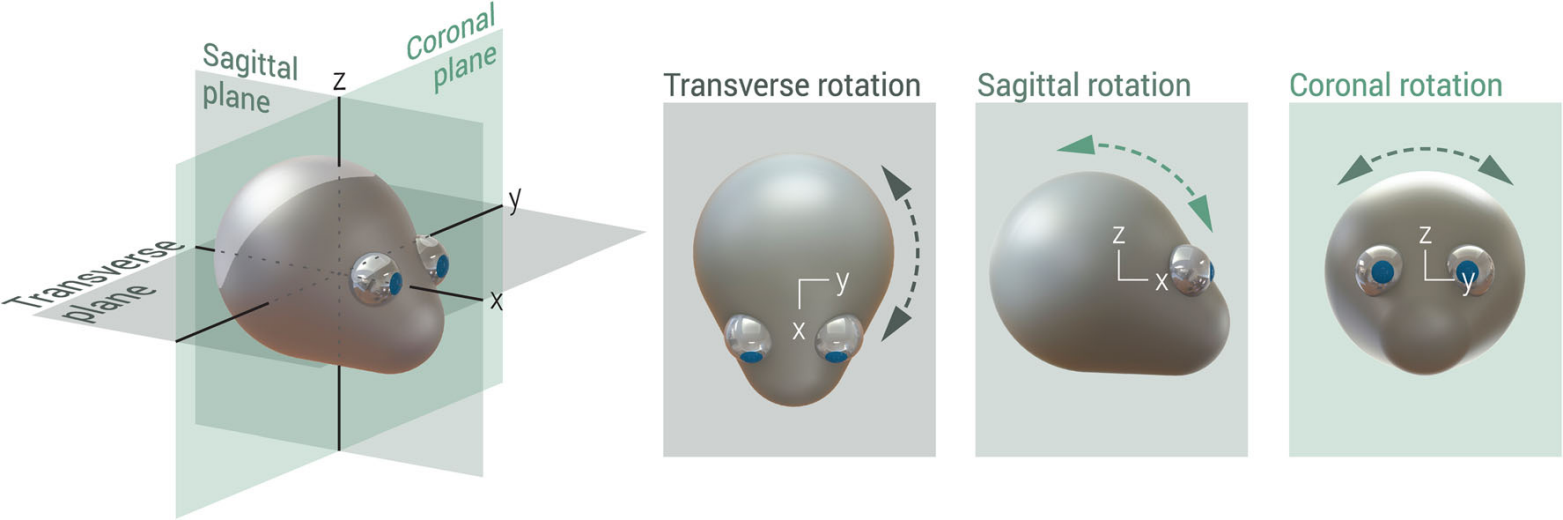
Rotational planes in animal studies of IHI-ST

The Margulies-group published nine articles, all of individual studies with 3-to-5-day-old piglets, apart from the 4-week-old piglets of Ibrahim et al. [21–29]. See Tables 3, 4, and 5 for more extensive details of the respective articles. Naim et al. injected half of their tested piglets with folic acid, which is beyond the scope of this review and data pertaining to that part of the study will hence not be included in this review [25]. In all pig studies, the animals were secured to a bite plate or padded snout clamp and moved in a single plane (Fig. 3). All but Coats et al. [27, 29] and Eucker et al. [28], rotated solely in the transverse (also referred to as axial) plane (Table 5) [21–26]. Head movements were applied as single, consecutive/double-single, or continuously repeated rotations. Macroscopy and microscopy (HE-staining, β-APP, NF68, NF200, and/or avidin-biotinperoxidase (ABC) histochemistry) was performed in all studies, although not on all brains. Furthermore, cervical spinal cords were examined by some, but reported on by none.

**Table 3:** Study design of piglet articles with shaking solely in a transverse plane (often referred to as axial in the articles) [6–10]

Article	Objective	Animals	Trauma mechanism	Fixation	Outcome measures	Input dynamics
Raghupathi 2002 [6] *Journal of Neurotrauma* Traumatic Axonal Injury after Closed Head Injury in the Neonatal Pig	To better understand the mechanical environment associated with closed pediatric head injury, by animal models including salient features.	7 piglets + 1 control; 3-to-5-day-old; average weight: 2.0 kg (1.5–3.0, 3 unknown). Average brain weight: 35 g (33–38).	Anesthesia and ventilation. Rapid, inertial, non-impact, transverse head rotation 110° over 10–12 ms, centered in the cervical spine, with HYGE pneumatic actuator. Heads secured to padded snout clamp.	6–8 h anesthesia and ventilation before death. Heparin perfusion, in situ fixation with 10% formalin, followed by ex-situ fixation overnight.	Macroscopy and microscopy of brain, cerebrum, and brain stem with Nissl staining, NF68 and NF200 immunohistochemistry; ABC-histochemistry.	Angular velocity of 272 rad/s. Average PAV of 250 ± 10 rad/s.
Raghupathi 2004 [7] *Journal of Neurotrauma* Traumatic axonal injury is exacerbated following repetitive closed head injury in the neonatal pig	To evaluate the effect of reducing the loading conditions on the extent of regional traumatic axonal injury, and to develop a model of repeated mild brain trauma.	11 piglets + 3 controls; 3-to-5-day-old. Group 1 (*n* = 5): single rotation (15 ms), ± weight 2.0 kg (1.8–2.4), ± brain weight 36 g. Group 2 (*n* = 6): double rotation (15 ms, 10–15 m apart), ± weight 2.1 kg (1.7–2.5), and ± brain weight: 35 g.	Anesthesia and ventilation. Rapid, non-impact, transverse rotations of the head centered in the cervical spine, with HYGE pneumatic actuator. Heads secured to padded snout clamp.	6 h Anesthesia and ventilation before death. Heparin perfusion, in situ fixation with 10% formalin, followed by ex-situ fixation overnight.	Macroscopy and microscopy of brain, cerebrum, and brain stem with NF200 immunohistochemistry, and ABC-histochemistry.	PAV averaging 172 rad/s for single and 138 rad/s for double loads.
Friess 2007 [8] *Experimental Neurology* Neurobehavioral Functional Deficits Following Closed Head Injury in the Neonatal Pig	To develop reliable quantitative functional neurobehavioral assessments for brain injury in piglets.	18 piglets + 9 controls; 3-to-5-day-old. Group 1 (*n* = 10): 1 moderate acceleration (188 rad/s). Group 2 (*n* = 5): controls moderate group. Group 3 (*n* = 8): 2 consecutive transverse, mild accelerated (142 rad/s) head rotations, 3.1 ± 0.5 min apart. Group 4 (*n* = 4): controls mild group.	Anesthesia and ventilation. Single, rapid, non-impact, transverse head rotation with the HYGE pneumatic actuator, 1–3 min after end of isoflurane. Heads secured to padded bite plate.	After 12 days re-anesthetized, death by pentobarbital, heparin and then in situ fixed with 10% formalin. Ex situ fixed overnight.	Macroscopy and microscopy of brain, cerebrum, brain stem, and high cervical spinal cord with HE staining, β-APP staining, and NF68 immunohistochemistry and counterstained with Meyer’s hematoxylin.	Moderate acceleration: 62.90 ± 10.10 krad/s^2^, velocity: 188 ± 7 rad/s. Mild acceleration: 34.12 ± 2.80 krad/s^2^, velocity: 142 ± 2 rad/s.
Friess 2009 [9] *Journal of Neurotrauma* Repeated traumatic brain injury affects composite cognitive function in piglets	To develop a cognitive composite dysfunction score to correlate white matter injury severity in piglets with neurobehavioral assessments.	21 piglets + 7 controls (7 littermate groups, of 5 piglets); 3-to-5-day-old. Group 1 (*n* = 7): single. Group 2 (*n* = 7): double; 1 day apart. Group 3 (*n* = 7): double; 7 days apart. Group 4 (*n* = 7): controls. Group 5 (*n* = 5): controls for group 3	Anesthesia and ventilation. Moderate (190 rad/s) rapid, non-impact, transverse angle rotation of 110° over 10–12 ms with HYGE pneumatic actuator. Heads secured to padded bite plate.	After 12 days re-anesthetised, death by pentobarbital/heparin, then in situ fixed with 10% formalin. Ex situ fixed overnight. Group 3 and 5 sacrificed after 5 days instead of 12.	Macroscopy and microscopy of brain, cerebrum, brain stem, and high cervical spinal cord with HE staining, β-APP staining, and counterstained with Meyer’s hematoxylin.	Velocity: Gr 1:: 193.7 rad/s, Gr 2: 196.7–195.9 rad/s, Gr 3:: 190.3–187.6 rad/s Acceleration: Gr 1: 58.51 krad/s^2^. Gr 2: 55.17–54.35 krad/s^2^. Gr 3: 57.32–56.12 krad/s^2^
Naim 2010 [10] *Developmental Neuroscience* Folic Acid Enhances Early Functional Recovery in a Piglet Model of Pediatric Head Injury	To test if folic acid supplementation after injury would decrease the severity of TAI in our well-established piglet model of moderate pediatric head injury.	4 groups: 40 female + 10 male piglets, 3-to-5-day-old. Group 1 (*n* = 7): injured + daily intraperitoneal folic acid injection (IF) 2.24 kg. Group 2 (*n* = 8): injured + daily saline injection (IS) 2.01 kg. Group 3 (*n* = 8): uninjured + daily folic acid injection (UF) 1.8 kg. Group 4 (*n* = 7): uninjured + daily saline injection (US) 1.99 kg. Group 5: behavior controls.	Anesthesia and ventilation. Rapid, inertial, 90–110° transverse rotation, centered in the cervical spine with the HYGE pneumatic actuator. Heads secured to padded bite plate.	After 6 days re-anesthetized, death by pentobarbital, heparin and then in situ fixed with 10% formalin.	Behavioral testing on days 1 and 4 following injury. Macroscopy and microscopy of brain, cerebrum, brain stem, and high cervical spinal cord with HE staining, β-APP staining, and counterstained with Meyer’s hematoxylin.	Angular velocity: IF group: 193.29 ± 5.31 rad/s, IS group: 194.25 ± 8.11 rad/s

β-APP immunohistochemistry (β-amyloid precursor protein) *HE-stain* hematoxylin and eosin stain, *NF* Neurofilament *ABC* avidin-biotinperoxidase histochemistry, *PAV* peak angular velocity

**Table 4:** Study design of 4-week old piglet article [11]

Article	Objective	Animals	Trauma mechanism	Fixation	Outcome measures	Input dynamics
Ibrahim 2010 [11] *Journal of Neurotrauma* Physiological and pathological responses to head rotations in toddler piglets	To characterize the physiological and pathological responses of the immature brain to inertial forces and their relationship to neurological development.	13 female piglets; brain weight 56.04 g, 4-week-old. Group 1 (*n* = 2): controls, Group 2 (*n* = 4): low rate angular acceleration, Group 3 (*n* = 6): moderate rate angular acceleration.	Anesthesia and ventilation. Single non-impact, transverse rotation, centered in the cervical spine. Heads secured to padded bite plate with snout straps and pneumatic actuator.	Euthanized 6 h after injury. Death by pentobarbital, in situ perfusion fixation with 10% formalin. Ex situ fixed in 10% formalin.	Macroscopy and microscopy of brain, cerebrum, brain stem, and high cervical spinal cord, with HE-staining, β-APP staining, NF68 and counterstained with Meyer’s hematoxylin.	Acceleration: low (31.6 ± 4.7 krad/s^2^,) or moderate (61.0 ± 7.5 krad/s^2^,). PAV: low: 129 ± 13 rad/s, moderate 194 ± 15 rad/s.

*β-APP* β-amyloid precursor protein, *HE-stain* hematoxylin and eosin stain, *NF* Neurofilament, *PAV* peak angular velocity

**Table 5:** Study design of piglet articles with movement in multiple planes [12–14]

Article	Objective	Animals	Trauma mechanism	Fixation	Outcome measures	Input dynamics
Coats 2010 [12] *Investigative Ophtalmology & Visual Science* Ocular Haemorrhages in Neonatal Porcine Eyes from Single, Rapid Rotational Events	To characterize ocular hemorrhages from single, rapid head rotations in the neonatal pig.	51 piglets + 5 controls; 3-to-5-day-old. Group 1 (*n* = 13): sagittal rotation, Group 2 (*n* = 7): coronal rotation, Group 3 (*n* = 31): transverse rotation, Group 4 (*n* = 5): controls.	Anesthesia and ventilation. Single rapid (15 ms), non-impact head rotation, centered in the C3-C5 spine, with HYGE pneumatic actuator. Heads secured to padded snout clamp.	6 h anesthesia before death by heparin infusion and in situ fixation with 10% formalin. Ex situ fixed overnight.	Brain macroscopy (46/51 animals), microscopy (31/51): brain, cerebrum, brainstem: HE-staining and NF68 or APP. Indirect ophthalmoscopy, (10 injured +2 controls) and macroscopy, microscopy (HE staining)	Angular velocities and accelerations: 117–266 rad/s and 30.6–101 krad/s^2^.
Eucker 2011 [13] *Experimental Neurology* Physiological and histopathological responses following closed rotational head injury depend on direction of head motion	The effect of sagittal and coronal rotation on regional cerebral blood flow changes, unconsciousness times, and apnea incidences, as well as early pathological outcomes.	36 piglets; 3-to-5-day-old. Group 1 (*n* = 9) HOR-HIGH: > 90° horizontal (transverse) rotation, Group 2 (*n* = 7) COR: > 90° coronal rotation, Group 3a (*n* = 6) SAG: > 60° sagittal rotation, Group 3b (*n* = 6) HOR-LOW: 90° horizontal (transverse) rotation, Group 4 (*n* = 4): controls.	Anesthesia and ventilation. A single rapid (12–20 ms), non-impact head rotation, centered at the mid-cervical spine with a bite plate.	Euthanized 6 h after injury. Death by pentobarbital, perfusion fixation/in situ. Fixation with 10% formalin. Ex situ fixed in 10% formalin for over 24 h.	Macroscopy and microscopy of brain, cerebrum, brain stem, and high cervical spinal cord, with HE-staining, β-APP staining and counterstained with Meyer’s hematoxylin.	Group 1: PAV of 198 ± 12 rad/s. Group 2: PAV 208 ± 11 rad/s. Group 3a: PAV 166 ± 3 rad/s. Group 3b: PAV 168 ± 3 rad/s. Group 4: (controls) 0 rad/s.
Coats 2017 [14] *Journal of Neurotrauma* Cyclic Head Rotations Produce Modest Brain Injury in Infant Piglets	To systematically investigate the post-injury pathological time course after cyclic low-velocity head rotations and compare them with single head rotations.	50 piglets + 4 controls; 3-to-5-day-old. Group A (*n* = 5): sagittal, episodic. Group B (*n* = 6 sagittal, 2 transverse): continuous 30 s. Group C (*n* = 4): transverse, continuous 10 s. Group D (*n* = 8): transverse, continuous 30 s. Group E (*n* = 9): transverse, double continuous 30 s. Group F (*n* = 5): transverse, continuous 30 s. Group G (*n* = 5): sagittal, single-noncyclic. Group H (*n* = 6): sagittal, single-noncyclic. Controls: (*n* = 2) 6 h + (*n* = 2) 24 h post-injury.	Anesthesia and ventilation. Non-impact, 30° sagittal or 50° transverse rotations of the head, centered in the cervical spine, with HYGE pneumatic actuator. Heads secured to bite plate; hyperflexion/ extension of the neck was avoided.	Sacrificed 6 h (Group A, B, G, and Controls), 24 h (Group C, D, E, H and Controls) or 6 days (Group F) after last injury.	Macroscopy and microscopy of brain, cerebrum, and brain stem; HE-staining, β-APP staining with Mayer Hematoxylin counterstaining. Eyes: Indirect fundus examination, macroscopy, and HE-staining.	Single axis angular rate transducer; 2–3 Hz. Sagittal: peak-to-peak average angular velocity (unclear how this was determined) 22.71 ± 3.49 rad/s and average peak angular acceleration of 606.21 ± 160.30 rad/s2. Transverse: peak-to-peak angular velocity 28.92 ± 2.85 rad/s and peak angular acceleration 780.08 ± 118.03 rad/s2.

*β-APP* β-amyloid precursor protein, *HE-stain* hematoxylin and eosin stain, *NF* Neurofilament, *PAV* peak angular velocity

No unintended mortality or morbidity was described in five of the nine piglet articles (Tables 3, 4, and 5) [21, 22, 26–29]. Friess et al. did not find mortality either but excluded 3 piglets from their ‘moderate acceleration’ group (62.9 krad/s^2^) (2 palate fractures and 1 with inability to feed), see Table 3 [23]. A significantly higher mortality (43%) was reported by Friess et al. for the double rotation, 1-day apart group (average 55.2 and 54.3 krad/s^2^ for respectively the first and second rotation) (1 apnea, 1 poor neurological outcome, and 1 unknown), compared to animals in which a single rotation was applied (average 58.5 krad/s^2^) (1 with poor neurological outcome, *p* < 0.05) (Table 3) [24]. Three additional animals were sacrificed because of palate fractures (2 from the ‘single rotation’ group, 1 from the ‘1-week apart’ group). In the study of Naim et al. 7 injured piglets died: 2 of hard palate fractures, 1 apnoea/cervical spine hematoma, and 3 large SDHs [25].

Duration of unconsciousness was reported in four articles. Raghupathi et al. did not find overt or extensive loss of consciousness in their injured piglets [21]. Their double rotation, 1-day apart group (average 55.2 krad/s^2^ and 54.3 krad/s^2^ for respectively the first and second rotation) (Table 3) had significantly longer unconsciousness durations than controls on day 0 (10.1 ± 3.4 SD vs. 2.8 ± 0.7 SD min) in the study of Friess et al. [24]. On day 7, the 1-week apart group (average 57.3 and 56.1 krad/s^2^ for respectively the first and second rotation) also had significant longer unconsciousness durations than controls (5.1 ± 0.7 SD vs. 2.8 ± 0.7 SD min). In the study of Naim et al. (Table 3) unconsciousness durations were significantly longer in the injured group (6.27 ± 0.1 SD min) compared to the control group (3.58 ± 0.1 SD min; *p* = 0.01) [25]. Furthermore, all piglets with a moderate-acceleration (average 61.0 krad/s^2^) injury of Ibrahim et al. were apneic post-injury, compared to 50% of the low-accelerated animals (average 31.6 krad/s^2^) and 0% of controls (*p* < 0.05) (Table 4) [26].

Ocular examinations were reported on by Coats et al. [27, 29]. In the 51 injured piglets (average 30.6 krad/s^2^) studied by Coats et al. in 2010 [27] ocular hemorrhages were found in 73% of these 51 piglets, of which 51% were bilateral and primarily located near the vitreous base. In cases with bilateral SDHs 26 (68%) had ocular hemorrhages, compared to one in a unilateral SDH case. All but two animals with ocular hemorrhages had brain injury. In their study from 2017Coats et al. [29] found no ocular injuries at all, possibly explained by a five-times lower rotational velocity compared to other studies e.g. Coats et al. 2010 [27] (Table 5).

Friess et al. [24] and Naim et al. [25] did not report any axial or extra-axial hemorrhages, which were reported in all other studies (Table 6). In general, hemorrhages are more frequent and more severe with increasing force, duration, or repetition. Coronal rotations had less frequent, and less severe hemorrhages and axonal injury (AI) at microscopy in Coats et al. and Eucker et al. compared to sagittal or transverse rotations (Table 6) [27, 28]. Eucker found a significantly higher subarachnoid hemorrhage (SAH) score for high velocity, transverse (in the article referred to as horizontally) rotated piglets and sagittal rotated piglets compared to controls. Extra-axial hemorrhages were mainly located frontally in the Raghupathi articles [21, 22].

**Table 6:** Outcomes in piglet studies [6–14]

Article	Group	Intervention	No piglet	angular velocity rad/s (± SD)	angular acceleration krad/s2 (± SD)	SDH (SAH)	PH	Ischemia	AI (β-APP, NF68, or NF200)
Raghupathi 2002 [6]: 7 piglets + 1 control; 3-to-5-day-old, 6–8 h till death^a^									
1		single rapid transverse rotation	7	250 (± 10)	116.70 (± 21.18) ^b^	+ (+)	+		4.5–8.7 axons/ mm^2^
2		control	1	0	0	- (−)	–		–
Raghupathi 2004 [7]: 11 piglets + 3 controls; 3-to-5-day-old, 6 h till death ^a^									
1		single transverse rotation	5	172 (± 17)	50.84 (± 5.56) ^c^	60% (−)	–		80%
2		double transverse rotation, 10–15 ms apart	6	136 (± 8) and 140 (± 6)	34.38 (± 8.88) and 35.98 (± 7.03) ^c^	100% (−)	–		83.3%
3		controls	3	0	0	- (−)	–		–
Friess 2007 [8]: 18 piglets + 9 controls; 3-to-5-day-old, 12 days till death									
1		single moderate transverse acceleration 188 rad/s	10 (3 excl)	188 (± 7)	62.90 (± 10.1)	- (100%)	10%		50%
2		controls moderate group	5			- (−)	–		–
3		2 consecutive rapid transverse acceleration 142 rad/s, ± 3.1 min apart (mild)	8	142 (± 2)	34.12 (± 28.0)	- (−)	–		–
4		controls mild group	4	0	0	- (−)	–		–
Friess 2009 [9]: 21 piglets + 7 controls; 3-to-5-day-old, 5 days or 12 days till death									
1		single transverse injury, 12 d survival	7 (3 excl)	193.7	58.51			1 severe, 1 moderate	0.07% (2 brain stem)
2		double transverse injury 1 d apart, 12 d survival	7 (3 excl)	196.7 and 195.9	55.17 and 54.35			2 severe	0.36% (3 brain stem)
3		double transverse 7 d apart, 5 d survival	7 (1 excl)	190.3 and 187.6	57.32 and 56.14				0.37% (1 brain stem)
4		controls, 12 d survival	7	0	0			1 moderate	–
5		single transverse injury controls, 5 d survival	5	192 (± 1)	52.55 (± 1.74)				0.25% total brain (*p* < 0.03 vs group 1)
Naim 2010 [10]: 40 piglets + 10 controls; 3-to-5-day-old, 6 days till death^d^									
2		single transverse injury + daily intraperitoneal saline injection (IS)	8	194.25 (± 8.11)					0.18% (*p* < 0.02)
4		uninjured + daily saline injection (US)	7	0					0.003% (*p* = 0.003)
5		controls	10	0					
Ibrahim 2010 [11]: 10 piglets + 2–3 controls; 4-week-old, 6 h till death ^a^									
1		controls	2–3?	0	0				
2		single transverse injury, low rate	4	128.5 (± 12.6)	31.6 (± 4.7)				
3		single transverse injury, moderate rate	6	194.0 (± 14.8)	61.0 (± 7.5)	*P* < 0.05 more than controls or low group		*P* < 0.05 more vs low group or controls	*P* < 0.05 more than control or low group
Coats 2010 [12]: 51 piglets + 5 controls; 3-to-5-day-old, 6 h till death									
1		single sagittal injury	13	185 (± 17)	30.6–101	100% bilateral	57%	x	71% diffuse, 14% focal
2		single coronal injury	7	208 (± 11)	30.6–101	0% bilateral, 71% unilateral	0%		0% diffuse, 20% focal
3		single transverse injury	31	207 (± 31)	30.6–101	96% bilateral	58%		53% diffuse, 32% focal
4		controls	5	0	0	–	–		–
1–3		overall results				83% bilateral, 11% unilateral		48%	
Eucker 2011 [13]: 29 piglets + 4 controls; 3-to-5-day-old, 6 h till death ^a^									
1		single horizontal (transverse) high velocity	9	198 (± 12)		(100%^e^)		56%	100%^e^
2		single coronal injury	7	208 (± 11)		29%		0%	14%
3a		single sagittal injury	6	166 (± 3)		(100%^e^)		83%^e^	100%^e^
3b		single horizontal (transverse) low velocity	7	168 (± 3)		(83%^e^)		33%	100%^e^
4		controls	4	0		(0%)		0%	25%
Coats 2017 [14]: 50 piglets + 4 controls; 3-to-5-day-old, 6 h, 24 h or 6 days till death ^f,g^									
A		sagittal episodic 6 h survival	5	22.96 (± 2.61)	606.21 (± 160.3)	20% (SDH + SAH)			0%
B		sagittal 30 s continuous 6 h survival	6	22.51 (± 4.33)	606.21 (± 160.3)	33% (SDH + SAH)			17%
B		transverse 30 s continuous 6 h survival	2	28.52 (± 4.05)	780.08 (± 118.03)	0% (SDH + SAH)			0%
C		transverse 10 s continuous 24 h survival	4	30.86 (± 0.77)	780.08 (± 118.03)	50% (SDH + SAH)			100%
D		transverse 30 s continuous 24 h survival	8	28.54 (± 2.67)	780.08 (± 118.03)	50% (SDH + SAH)			25%
E		transverse double continuous 24 h survival	9	28.75 (± 3.02)	780.08 (± 118.03)	67% (SDH + SAH)			56%
F		transverse 30 s continuous 6 d survival	5	28.41 (± 3.87)	780.08 (± 118.03)	40% (SDH + SAH)			80%
G		sagittal noncyclic 6 h survival	5	32.19 (± 7.04)	2857.40 (± 1682.91)	0% (SDH + SAH)			0%
H		sagittal noncyclic 24 h survival	6	42.86 (± 6.45)	866.33 (± 213.92)	0% (SDH + SAH)			33%
Sham		controls 6 h and 24 h survival	2 / 2	0		0% (SDH + SAH)			0%

*SDH* subdural hematoma, *SAH* subarachnoid hemorrhage, *PH* parenchyma hemorrhage, *AI* axonal injury

^a^peak angular velocities instead of angular velocities, ^b^deceleration instead of acceleration, ^c^maximal deceleration instead of acceleration, ^d^Because of intervention piglets injected with folic acid are not representable for IIHI in human infants, and thus excluded from this table, ^e^significant, ^f^peak-to-peak angular velocities, ^g^angular acceleration in rad/s^2^ instead of krad/s^2^. NB: not all brains were macroscopically and/or microscopically examined

The time between two injuries and the time between injury and measurement might be of influence on the extent of measured AI, according to Friess et al. and Coats et al. [24, 29]. Single rotated piglets surviving 12 days (average 58.5 krad/s^2^) had significantly less β-APP staining (sign of AI: 2 h to 4–6 weeks) compared to single rotated piglets surviving 5 days (*p* < 0.03), thus less white matter injury was detected over time [24]. Episodic and continuous cyclic head rotations for 30 s had no differences in the amount of AI after 6 h [29]. There was a significant increase in AI with increasing post-injury time (24 h vs. 6 h) for 30 s continuous rotated animals (*p* = 0.035).

No AI was found in the controls. AI was found in all studies to some extent in injured groups, although not always significantly different from the control groups. Raghupathi et al. found no neuronal loss, nor a relation between the velocity and density of AI located in multiple white matter tracts [21]. In 2004 they reported that two consecutive rotations caused AI in the peripheral subcortical and central deep white matter regions, especially more foci with multiple injured axons (*p* = 0.05) compared to a single rotation [22]. After a single rotation, AI was found in the peripheral subcortical and central deep white matter regions in the frontal lobes. After two consecutive rotations, AI was also present in the white matter of the parietal and temporal lobes, corpus callosum, hippocampus, and basal ganglia. Friess et al. found AI in the olfactory tract, germinal matrix, internal capsule, and some posteriorly in the moderately rotated piglets (average 62.9 krad/s^2^), compared to no AI in the mildly rotated group (average 34.1 krad/s^2^) (Tables 3 and 6) [23]. In 2009 they reported that the majority of AI was located in the frontal lobes in injured animals with a significantly greater white matter β-APP injury volume in all three injury groups compared to uninjured animals [24]. Naim et al. found white matter injuries mostly in the deep white matter of the frontal lobes and some in parietal and temporal lobes or brainstem [25]. Eucker et al. found a significantly greater early ischemia score in the sagittal rotations than in controls [28]. High velocity transverse rotations resulted in significantly more AI than coronal or low-velocity horizontal rotations (Table 5). Both sagittal and transverse rotations produced the greatest degrees of tissue pathology, whereas coronal rotations did not result in any significant pathology. AI was more extensive in the anterior regions of the brain compared to other brain regions for every injury group, after multiple regression analysis. Coats et al. found injury in 88.5% of the cyclic rotated animals surviving 24 h and 6 days [29]. After 24 h there were significantly more animals with AI after continuous rotations for 10 s than 30 s (*p* = 0.014). There was more hypoxic-ischemic injury and extra-axial hemorrhages in 30 s continuous rotated piglets than in piglets with a single head rotation, but this was only noticeable after 24 h.

Ibrahim et al. compared the result of 4-week-old piglets to the previously published results by Eucker et al. in 5-day-old piglets [26, 30]. They found no significant differences in SAH scores and brain volume of AI between those two groups. However, when comparing these 4-week-old animals to 5-day-old piglets, based on the mass scaled acceleration principle of Ommaya and Hirsch, 1971 [31], there was a significant difference for both SAH scores (*p* < 0.02) and brain volume of AI (*p* < 0.004). In comparison to lower rotational acceleration (average 31.6 krad/s^2^), larger rotational accelerations (average 61.0 krad/s^2^) caused more severe SAH, more areas of ischemia, and larger volumes of AI.

## Discussion

Ommaya was the first to describe brain injury after a whiplash trauma in primates with SDH in 15 out of 19 monkeys with a concussion [14, 32, 33]. IHI-ST is, as stated before, still an important and current topic of debate. It might be expected that this would be reason for extensive studies in animals in the past by many different research groups. However, in this systematic review, only two research groups had usable articles. Older articles from 1998, 2002, and 2004 in rodents were excluded for their inconclusive reporting of methodology and results [12, 13, 16, 17]. Though Ommaya was the first to describe these kinds of injuries, the used trauma mechanism is incomparable to that of IHI-ST, why these studies were also excluded. The included piglet and lamb articles are difficult to compare because of differences in species, age, trauma mechanisms, and small study groups without source data available. With increasing moral and ethical standards, animals are less and less used as study objects and replaced by mathematical and physical models as described in Part II of this review. These two combined systematic reviews are the novel for IHI-ST.

### Lambs

IHI-ST, as described earlier, is most closely resembled by the study with shaken lambs. Like in human babies, the lambs were held by adults around the ribcage leaving the head free for acceleration-deceleration rotation in any direction for a significant amount of time (30 s) without a direct impact trauma. Accelerations generated in lambs by shaking have been reported by Sandoz et al. and Anderson et al. [11, 15]. Although the articles state that the lambs were shaken for 20 s, additional information by the main author confirmed that the shaking was actually for 30 s as in the three Finnie articles. Anderson added that three adults manually shook the animals, mainly in the sagittal plane. Shaking speed and perimeter differed per person and weight of the animals. Forces were measured with a triaxial piezoresistive accelerometer (8 g, 2000 Hz, model 7268C, Endevco©) and a motion tracking sensor (9.1 g, 60 Hz, Fastrak-Polhemus©) on the head and one motion tracking sensor under the axilla. Animals were shaken with approximately 2 Hz, thus around 40 cycles per shaking episode [11]. Accelerations (in absolute value, not otherwise explained) were between 0 and 5 g in 94.43% of cases, with a maximum of 26.64 g [15]. Acceleration of impulses >30 g had peak measurements of 58-79 g and average peak accelerations of 35.9–41.6 g for the younger animals, and 39-80 g and 34.1–44.9 g, respectively, for the older lambs.

The lambs were compared to 9-month-old human infants by the authors, based on their body weight. The pathophysiology of trauma due to shaking was deemed comparable to human infants since both have weak neck muscles, though this effect might have been exaggerated by the anesthesia in the lambs. Both human infants and lambs have a relatively large head/brain compared to the body, along with a wide subarachnoid space, allowing a relatively large brain movement within the skull [34]. Both have brains that are not yet fully myelinated, with a higher brain water content, and thereby an increased vulnerability to shearing injury [9, 35]. This may explain the more extensive injuries in the younger lambs compared to the older lambs (Table 2). The lamb brain is more elliptically shaped and is in line with the myelum and cervical spinal cord compared to an almost 90-degree angle between the rounder human brain and the spinal cord. The effect of the difference in shape and orientation of the brain is not known. A drawback of these publications on lambs is the fact that, according to the main author, all three articles were based on the same nine injured and four control lambs. Results therefore should be reproduced by other and larger studies.

### Piglets

In all piglet studies, except Coats et al. [29], only single accelerations were applied to the study animals. This reduces its value for translation to human shaken babies as it is believed by many that the repeated sudden deceleration, in combination with the acceleration, causes the intracranial injury [34]. When shaking a human baby, the head will rotate mostly in a sagittal plane, but may sustain rotations in the transverse and coronal planes, along with possible chin-chest collisions, depending on body weight and individual shakers [11]. Inconsistency in the use of terminology for rotation directions hinders interpretation and translation to humans. Different rotation planes have been studied by Coats et al., Eucker et al., and Coats et al. [27–29]. However, none of these studies combined the different rotation planes, while simultaneously occurring rotations in different directions might amplify the forces and deformations exerted on the anatomical structures in the head and hence worsen the resulting injuries. Furthermore, chin-chest collisions were avoided within these studies. More importantly, the single plane movement (only transverse in 6 out of 9 piglet studies) thus does not represent the main repeated back-and-forth motions and internal translation of forces in IHI-ST. Therefore, there are insufficient data to estimate whether and to what extent the results of these studies could be translated to IHI-ST.

Though executed and published by a single research group, specific reports on physics are inconsistent. Angular velocities are reported by Friess et al. [23, 24], Naim et al. [25] and Coats et al. [27] compared to peak angular velocities by Raghupathi et al. [21, 22], Ibrahim et al. [26] and Coats et al. [29], and even peak-to-peak angular velocities by Coats et al. [29]. Where most articles report on angular accelerations, Raghupathi et al. report on decelerations in 2002 and maximum decelerations in 2004. Furthermore, most reported angular accelerations were in krad/s^2^ ranges, such as Coats et al. [27], who reported 30.60–101 krad/s^2^ for angular velocities of 177–266 rad/s. These high angular velocities do, depending on the angle over which the head was moved (which is, unfortunately, not in all articles reported), resemble shaking with frequencies of 20 to 161 Hz, assuming angular ranges of motion of 110 to 30 degrees.

Shaking in human infants is often not a onetime occurring incident in time, but an event reoccurring over longer periods of time. Friess et al. and Raghupathi et al. postulated that repeating of the trauma within 24 h might increase the sensitivity to injury by latent readjustment or injury accumulation [22, 24]. Besides the aforementioned influences of anesthesia, buprenorphine and isoflurane anesthetics used in the piglet studies might have some neuroprotective features and could affect the results by reducing the injury [36, 37]. Mortality was reported in none of the articles. Friess et al. excluded some piglets because e.g. palate fractures or an inability to being fed due to the injury procedure. Because of exclusion those piglets were not counted as a mortality [23, 24].

Like humans, pigs have a gyrencephalic brain, with similar grey-white differentiation and physiological responses, and are therefore commonly used as a model for human infants. A 3-to-5-day-old piglet brain can be roughly compared to a 2- to-4-weeks-old human baby based on activity, myelination, and growth. Ibrahim et al. state that a 4-week-old piglet brain is comparable to a 2- to-4-year-old human brain based on development and myelination. Like the lamb brain, the piglet brain is more elliptically shaped and angled in line with the myelum and cervical spinal cord, compared to an almost 90-degree angle between the rounder human brain and the myelum. The single plane rotational movement in the piglet studies and the unalike anatomy make the results of these studies difficult to translate to the human infant.

The piglet’s eye has more in common with the human eye than most other animals’ eyes, for example, the retina vascularization and the vitreous base, although there are also differences such as the absence of a fovea or macula [38, 39]. The acceleration-deceleration trauma in IHI-ST is thought by some to cause the vitreous body to pull on the retina and consequently induce vascular injury. Yet, due to all the differences, it is hard to estimate whether the results of Coats et al. [27] could be used for human injury assessment.

## Conclusion

Injury sustained by lambs in shaking studies gives some information on the relationship between the applied shaking accelerations, the animal, and the clinical outcome. Older, heavier lambs had less AI and deaths. For piglets, it was found that rotation direction influenced the neurological symptoms and neuropathological findings. Tissue strain might be of influence on these injuries, yet the anatomical differences and the inconsistent choice of (mostly noncyclic) rotation directions in the various studies make an adequate comparison very difficult. With regard to the ocular results in piglets, no direct translation to human infants can be made due to differences in anatomy and a lack of evidence of the relevance of these differences. The study in 4-week-old piglets might provide some information about single rotational impacts in toddlers, but lacks confirmation by other studies. Future studies should therefore focus on understanding each individual step in the IHI-ST process and its respective accelerations, forces, tissue deformations and injury thresholds. Ideally, experimental conditions should allow a free movement of the head in all directions, without any external impacts, in simulations comparable to inflicted head injury due to shaking in human infants.

## Key points

1. Despite the importance for understanding IHI-ST, adequate large randomized animal studies are lacking in the literature.
2. Animal articles, closely representative of IHI-ST as presumed to occur in human infants, are only available from a single study in lambs.
3. Studies in piglets provide some information on IHI-ST, mostly about a fast transverse acceleration, but are otherwise difficult to translate to human infants because of the (non-cyclic) rotational movement restrictions.
4. Future research should focus on larger, more consistent animal studies, validating the applicability of juvenile animal experiments as a model of human IHI-ST.

## Appendix 1 Search Queries

1. ((finite[All Fields] AND (“elements”[MeSH Terms] OR “elements”[All Fields] OR “element”[All Fields]) AND shaken[All Fields]) OR (((biomechanical[All Fields] AND shaken[All Fields]) OR ((“models, animal”[MeSH Terms] OR (“models”[All Fields] AND “animal”[All Fields]) OR “animal models”[All Fields] OR (“animal”[All Fields] AND “model”[All Fields]) OR “animal model”[All Fields]) AND (“shaken baby syndrome”[MeSH Terms] OR (“shaken”[All Fields] AND “baby”[All Fields] AND “syndrome”[All Fields]) OR “shaken baby syndrome”[All Fields] OR (“shaken”[All Fields] AND “baby”[All Fields]) OR “shaken baby”[All Fields]))) OR (non[All Fields] AND accidental[All Fields] AND (“craniocerebral trauma”[MeSH Terms] OR (“craniocerebral”[All Fields] AND “trauma”[All Fields]) OR “craniocerebral trauma”[All Fields] OR (“head”[All Fields] AND “injury”[All Fields]) OR “head injury”[All Fields]) AND model[All Fields]))) OR ((“Simulation”[Journal] OR “simulation”[All Fields]) AND shaken[All Fields])
2. (((((((biomechanical) OR animal model) OR finite element) OR simulation) OR mannequin) OR dummy)) AND (((shaken baby) OR abusive head trauma) OR non accidental head)
3. (((Biomechanical Phenomena/methods [Mesh]) OR (((((((biomechanical model) OR biomechanical evaluation) OR biomechanical study) OR biomechanical) OR biomechanical analysis) OR “Models, Neurological”[Mesh]) OR “Models, Theoretical”[Mesh]))) AND ((((((((((((Hematomas, Subdural) OR Subdural Hematomas) OR Subdural Hematoma) OR Hemorrhage, Subdural) OR Hemorrhages, Subdural) OR Subdural Hemorrhage) OR Subdural Hematoma, Traumatic) OR Subdural Hemorrhages) OR Hematoma, Traumatic Subdural) OR Hematomas, Traumatic Subdural) OR Traumatic Subdural Hematoma) OR Traumatic Subdural Hematomas)
4. (((biomechanic* OR dynamic* OR kinematic* OR motion OR force OR impact) AND (phenomena OR method OR model OR evaluation OR study OR analysis)) OR (“finite element” OR “FEM”) OR ((animal OR neurological OR theoretical) AND model) OR simulat* OR doll OR mannequin OR dummy OR anthropomorphic) AND ((shake* AND (infant OR baby OR impact)) AND (“subdural Hematoma” OR “subdural Hemorrhage” OR ((craniocerebral OR head OR retinal) AND (injury OR trauma OR bleeding))) AND ((“non accidental” OR “nonaccidental” OR “non-accidental”) OR inflict* OR violen* OR abus* OR shaking))

*Pubmed was searched using queries Q1 to Q4 and combining their results. Scopus was searched using query Q4.*
